# Obstetrical Statistics

**Published:** 1851-01

**Authors:** P. O. Williams

**Affiliations:** Gouverneur, N. Y.


ART. II.—*Obstetrical Statistics, reported by* DR. P. O. WILLIAMS, of Gouverneur, N. Y.

MR. EDITOR:—I send you the results of my observations in the obstetrical cases that have come under my notice for the past three years and six months, ending the 7th November, 1850, which you can publish if you think it proper to do so; not that there is any thing peculiar in my practice, or the report of my cases, but that a Physician's diary in this, and every other branch of the medical profession, is too little attended to for his own benefit, and that of his patients; and the reported cases far too few—which might, if it were otherwise, benefit the profession. Extensive and practical reports might be made by physicians of far more capability and experience than myself.

| | |
|---|---|
| Whole number of Cases, | 216 |
| " " boys, | 125 |
| " " girls, | 91 |
| Of these the whole number of abortions were, | 5 |
| " " " girls, | 3 |
| " " " boys, | 2 |
| Whole number of premature labors, | 10 |
| " " " " girls, | 6 |
| " " " " boys, | 4 |

| | |
|---|---|
| The greatest weight of any boy, | 14$\frac{3}{4}$ |
| The least, excluding abortions, | 5 |
| Average weight of boys, | 8$\frac{1}{2}$ |
| The greatest weight of any girl was, | 13$\frac{1}{2}$ |
| The least, excluding abortions, | 3 |
| Average weight of girls, | 7$\frac{1}{2}$ |
| Whole number of occiput presentatations, | 169 |
| " " " girls, | 70 |
| " " " boys, | 99 |
| Whole number of face presentations, | 20 |
| " " " girls, | 12 |
| " " " boys, | 8 |
| Whole number of breech presentations, | 12 |
| " " " girls, | 7 |
| " " " boys, | 5 |
| Whole number of presentations of inferior extremities, | 4 |
| " " " girls, | 3 |
| " " " boys, | 1 |
| Whole number of presentations of shoulder, | 3 |
| " " " girls, | 1 |
| " " " boys, | 2 |
| Whole number of presentations of superior extremities, | 2 |
| " " " girls, | 1 |
| " " " boys, | 1 |
| Whole number of placenta presentations (or placenta praevia), | 3 |
| " " " girls, | 2 |
| " " " boys, | 1 |
| Presentations of abortive cases not known. | |
| Whole number of cases of retained placenta, | 3 |
| " " " girls born, | 3 |
| Whole number of children who died just before, or just after the delivery of mother, not including abortives, | 6 |
| The causes of death were malformation of heart, | 1 |
| Unknown, | 2 |
| From injuries of mother, | 2 |
| Monstrosity, | 1 |

Brain and medulla spinalis exposed ; occiput spinous. Transverse and oblique processes wanting. Face resembles that of a toad ; weight 8$\frac{1}{2}$ pounds ; and makes a fine addition to my museum.

| | |
|---|---|
| The whole number of mothers that suffered from phlegmasia dolens, | 6 |
| One limb only, | 4 |
| Both limbs, | 2 |
| The whole number of cases of puerperal fever, | 3 |
| " " " peritonitis, | 2 |
| " " " hysteritis, | 2 |
| " " " " mania, | 1 |
| The whole number of cases of convulsions before birth of child, | 1 |
| " " " " " after birth of child, | 1 |
| The whole number of cases of excessive avoidable flooding, | 2 |

All the abortions were produced by a repeated use of emenagogues.

Of the whole number of cases one mother died ; cause, the absorption of effluvia from decomposed fœtus; inflammation of stomach, bowels, and uterus from repeated use of supposed abortives terminating in hectic and death.

| | |
|---|---|
| The least time that any woman was in labor, (I give the time that I was detained,) was, hours, | $\frac{1}{4}$ |
| The longest time, hours, | 36 |
| Average time, hours, | $3\frac{1}{4}$ |

In no case have I used an instrument, and in no case has the mother died, except the one above mentioned.

Gouverneur, November, 1850.

